# How to switch on a master switch

**DOI:** 10.1093/jxb/erae116

**Published:** 2024-05-20

**Authors:** Navneet Kaur, Nigel G Halford

**Affiliations:** Rothamsted Research, Harpenden, Hertfordshire AL5 2JQ, UK; Rothamsted Research, Harpenden, Hertfordshire AL5 2JQ, UK

**Keywords:** AMPK, compound 991, germination, phosphoproteomics, rice, SnRK1

## Abstract

This article comments on:

Hu Y, Lin Y, Bai J, Xu X, Wang Z, Ding C, Ding Y, Chen L. 2024. AMPK activator 991 specifically activates SnRK1 and thereby affects seed germination in rice. Journal of Experimental Botany 75, 2917–2932.

**Sucrose non-fermenting-1-related protein kinase (SnRK1) is a key regulator of metabolism in plants, with wide-ranging effects on carbon and nitrogen metabolism and the interplay between metabolic, stress, and developmental signalling. Modulating its activity either genetically or chemically has become a target for improving crop performance, making the identification of chemicals that affect SnRK1 activity extremely important. In their study, Hu *et al*. (2024) investigated the effect of compound 991, a cyclic benzimidazole derivative and direct activator of SnRK1’s mammalian counterpart, AMP-activated protein kinase (AMPK). They showed that addition of the compound not only increased SnRK1 activity in extracts from germinating rice seeds but also promoted germination at low concentrations. Moreover, they discovered that the phosphopeptides induced by compound 991 treatment overlapped with those induced by *OsSnRK1α* overexpression.**

SnRK1 can truly be described as a global regulator of carbon and nitrogen metabolism in plants, operating through the modulation of enzyme activity and gene expression, and sitting at the interface between metabolic, stress, and developmental signalling networks. It phosphorylates and inactivates key metabolic enzymes, such as HMG-CoA reductase, nitrate reductase, and sucrose phosphate synthase (Sugden *et al*., 1999), and is involved in regulating the expression of genes encoding α-amylase (Laurie *et al*., 2003; Lu *et al*., 2007) and asparagine synthetase (Baena-González *et al*., 2007). The authors have previously shown SnRK1 to be required for the remobilization of non-structural carbohydrates in rice sheaths during grain filling (Hu *et al*., 2022), and it is also involved in the re-allocation of carbon in response to herbivory (Schwachtje *et al*., 2006). SnRK1 also promotes starch accumulation in grains and tubers through modulation of sucrose synthase and ADP-glucose pyrophosphorylase gene expression (Kanegae *et al*., 2005; McKibbin *et al*., 2006) and ADP-glucose pyrophosphorylase redox activation (Tiessen *et al*., 2003). Null mutations of *SnRK1* in Arabidopsis are lethal (Baena-González *et al*., 2007), while *Physcomitrella patens* nulls can survive but only under constant illumination (Thelander *et al*., 2004). We do not have the space here to do justice to the body of work that has been produced on SnRK1, but refer the reader to a review provided by Emanuelle *et al*. (2016).

Like a number of proteins involved in the regulation of ancient signalling pathways that control fundamental cellular processes, SnRK1 shows a high level of conservation with its fungal and animal counterparts. These are sucrose non-fermenting-1 (SNF1) of fungi and AMPK of animals (Halford and Hardie, 1998). The plant, animal, and fungal protein kinases share 47% amino acid sequence identity and show similar heterotrimeric structures and substrate specificity, despite being evolutionally separated for 1–1.5 billion years. The similarities are so close that *SnRK1* will complement the *snf1* mutation of budding yeast (Alderson *et al*., 1991), enabling yeast cells lacking a functional *SNF1* gene to utilize alternative carbon sources to glucose when otherwise they would starve. However, that is not to say that the systems in fungi, plants, and animals are the same, with two differences that stand out, in particular. The first of these is the burgeoning of the *SnRK* gene family in plants and its divergence into three classes, with *SnRK1* joined by *SnRK2* and *SnRK3*, both of which have multiple members and are unique to plants (Halford *et al*., 2003). The second is the mode of activation: AMPK is allosterically activated by AMP, as well as by phosphorylation, which itself is stimulated by AMP (Hardie *et al*., 2012). AMP accumulation is a very good indicator of cellular energy stress (Hardie *et al*., 2012), but neither SNF1 nor SnRK1 is activated in the same way. Indeed, the metabolite or metabolites that are sensed by the SnRK1 system remained elusive for many years until trehalose 6-phosphate (T6P) was identified as a potent inhibitor (Zhang *et al*., 2009; Oszvald *et al*., 2018).

The identification of T6P as an inhibitor of SnRK1 led to its development as a biostimulant (Griffiths *et al*., 2016). There has been similar interest in factors that affect AMPK activity because of their potential in treating metabolic diseases, and compound 991 is one of the chemicals that has emerged from that work as an AMPK activator. Hu *et al*. (2024) showed that compound 991 will also activate SnRK1.

## Compound 991 is an activator of SnRK1 and modulator of seed germination in rice and wheat

Hu *et al*. (2024) measured SnRK1 activity in germinated rice seed extracts treated with varying concentrations (0.1–100 μM) of the compound and showed that SnRK1 activity increased by up to 75%. In rice, the application of low concentrations of compound 991 (ranging from 0.5 μM to 10 μM) also exhibited a positive effect on germination, while higher concentrations (100 μM) resulted in a pronounced reduction in germination rate. Root length, shoot length, and seedling weight increased under 0.5, 1, and 10 μM treatments relative to the control, whereas they decreased under the 100 μM treatment. These results demonstrate a dose-dependent modulation of rice seed germination by compound 991.

The team conducted parallel assays on wheat, employing the same concentrations of compound 991 as in the rice experiments. Germination was accelerated under 0.5 μM and 1 μM 991 treatments, but the 10 μM treatment delayed germination, with the delay being further intensified under the 100 μM treatment. The authors suggest that delayed germination under higher concentrations of compound 991 could have been due to disruptions of metabolism caused by excessive SnRK1 activity.

## Overexpression of *OsSnRK1α* induces a similar response to treatment with compound 991

Hu *et al*. (2024) developed four transgenic rice lines overexpressing *OsSnRK1α*, which encodes the catalytic subunit of SnRK1, and used them to investigate whether the impact of compound 991 on germination was attributable to increased SnRK1 activity. The lines exhibited 1.2- to 1.74-fold increases in *OsSnRK1α* expression. Three of the lines displayed accelerated germination, accompanied by increases in root length, shoot length, and seedling weight, but the line with the highest *OsSnRK1α* expression demonstrated reduced germination, root length, shoot length, and seedling weight. These effects were, therefore, very similar to those induced by treatment with compound 991.

## *SnRK1α* mutant insensitivity to treatment supports specific targeting of SnRK1 by compound 991

Hu *et al*. used a rice *snrk1α* mutant in which the catalytic subunit of the protein kinase lacks the C-terminal regulatory domain required for catalytic regulation and heterotrimeric complex formation. They compared the response of the *snrk1α* mutant and wild-type control to compound 991 treatment during germination. The *snrk1α* mutant showed no change in germination, root length or seedling weight when treated with 1 μM compound 991, apart from a modest increase in shoot length, whereas the wild-type control showed accelerated germination, increased shoot and root length, and increased seedling weight. This reduced sensitivity of the *snrk1α* mutant to the effects of compound 991 further supported the involvement of SnRK1 in mediating the impact of compound 991 on rice seed germination, as well as showing the importance of the SnRK1 regulatory domain for activation.

## Overlap of phosphopeptides induced by compound 991 treatment and *OsSnRK1α* overexpression

Phosphoproteomic analysis was performed on untreated seeds of wild-type plants, untreated seeds of an *OsSnRK1α*-overexpressing line (35S-8), and wild-type seeds treated with 1 μM and 100 μM compound 991 for 72 h. With respect to the untreated wild-type seeds, differentially expressed phosphopeptides (DEPs) were identified in the *OsSnRK1α*-overexpressing line and in the wild-type seeds after both treatments, with close similarities between the treated wild-type seeds and the untreated seeds of the overexpressing line (46.4% overlap for the 1 μM treatment, rising to 61.3% for the 100 μM treatment). Gene Ontology (GO) enrichment analysis also showed significant overlaps of 37.7% and 50.5% in enriched terms between the treated wild-type seeds and *OsSnRK1α*-overexpressing seeds, indicating that compound 991 activates the phosphorylation network regulated by SnRK1 during germination. Notably, up-regulated DEPs in the *OsSnRK1α* overexpression line outnumbered down-regulated DEPs by approximately three to one, consistent with SnRK1 being a central phosphorylation hub during rice germination.

This comprehensive approach allowed the identification of specific protein targets and pathways affected by SnRK1α activation, induced either by *OsSnRK1α* overexpression or by compound 991 treatment. It also helped to delineate the specific role of SnRK1α activation in mediating the cellular responses to compound 991. It is an excellent example of just how powerful a tool phosphoproteomics profiling can be in the elucidation of signalling networks and the roles of the protein kinases that control them.

## Phosphosites in PIP2, SOS1, and ABI5: critical for germination and regulated by SnRK1

To elucidate the mechanism underlying compound 991’s dose-dependent regulation of rice seed germination, and by inference the role of SnRK1, a time-series was compiled of phosphopeptides affected by treatment with 1 μM and 100 μM of the compound. Two clusters emerged, the first of which exhibited increased phosphorylation levels with higher 991 concentrations and included phosphosites on two plasma membrane intrinsic proteins, PIP2;4 and SOS1 (a plasma membrane Na^+^/H^+^ exchanger), both of which are crucial for seed germination. The second cluster included Ser110 of the abscisic acid (ABA) response element-binding protein, ABI5 (ABA-insensitive-5), a negative regulator of germination. The amino acid sequence around this serine is LPRTLSQKTV, which is a classic SnRK1 target site (a hydrophobic residue at –5 and +4 relative to the serine, a basic residue at –3). A peptide including this motif has been shown to be phosphorylated by SnRK1 *in vitro* (Zhang *et al*., 2008).

## Future prospects

In conclusion, the identification of compound 991 as an activator of SnRK1 is tremendously important because of the potential for using it to explore the function of what is a master metabolic switch that links plant metabolic regulation with stress and developmental signalling networks. Compound 991 was effective in wheat as well as rice, with germination in wheat appearing to be more sensitive than in rice, but it is important that it be tested in a wider range of plant species. It will also be fascinating to explore the interplay between compound 991, T6P, and SnRK1 and its effect on source–sink interactions and nutrient allocation (Griffiths *et al*., 2016; Oszvald *et al*., 2018; Hu *et al*., 2022). As with T6P, there may even be applications for compound 991 in plant growth regulation in agriculture.
